# *Tu quoque?!* On the second person pronoun *tusya* (*tus̱a*) and the second person verbal ending -*tu* (-*du*) in Niya Prakrit

**DOI:** 10.1515/jsall-2022-2044

**Published:** 2022-09-16

**Authors:** Niels Schoubben

**Affiliations:** Leiden University, Leiden, The Netherlands

**Keywords:** grammatical number, Niya Prakrit, pronominal morphology, tense, aspect and mood (TAM), verbal morphology

## Abstract

When compared to Old Indo-Aryan (OIA) and other Middle Indo-Aryan (MIA) languages, the marking of the second person in Niya Prakrit differs in two crucial respects. On the one hand, Niya Prakrit makes use of a pronoun *tusya*/*tus̱a* ‘you’ that is not found in the other languages. On the other hand, Niya Prakrit has a verbal ending -*tu* (-*du*) as a second person marker on top of the old 2sg ending -*si* (-*s̱i*) and the 2pl ending -*tha*. This paper argues that these two peculiarities are related to one another and that both the pronoun and the verbal ending have not been properly described in earlier scholarship. First, it will be claimed that *tusya* (*tus̱a*) is not a genitive singular (gen.sg), as previously thought, but a direct plural (dir.pl). As a consequence, a new etymology for this pronoun will be offered too. Second, this article presents various arguments that -*tu* (-*du*) is not a 2sg ending, but a 2pl.

## 1 Aims and methods

The present paper is concerned with the pronominal and verbal morphology of Niya Prakrit, i.e. the dialect of Gāndhārī used in administrative texts from the Late Antique Shanshan kingdom in present-day Xinjiang (Høisæter 2020; Padwa 2007). In line with the other Middle Indo-Aryan (MIA) languages, Niya Prakrit has three persons and two numbers (sg and pl), but the marking of the second person differs from the other Prakrits in two significant respects. At the pronominal level, Niya Prakrit makes use of the form *tusya*/*tus̱a* ‘you’, which seems to lack an exact counterpart in closely related languages. With regard to the verb, Niya Prakrit does not only use the inherited 2sg ending -*si* (-*s̱i*)1I list here an approximate phonetic interpretation of some Kharoṣṭhī akṣaras [graphemes] which occur in the present article and which readers used to Brāhmī-derived scripts may not be familiar with. ⟨g̱⟩ ∼ [ɣ]; ⟨j̱⟩ ∼ [ʝ]; ⟨ṭh⟩ ∼ [ʂʈ]; ⟨ḍ̱⟩ ∼ [ɽ]; ⟨v̱⟩ ∼ [w]; ⟨s̱⟩ ∼ [z]. It is commonly assumed that all intervocalic consonants were lenited to fricatives in Gāndhārī, as a result of which the frequently interchanging ⟨t⟩ and ⟨d⟩ probably stand for [ð] in intervocalic position. See Baums (2009: 110–200) for a detailed discussion on Gāndhārī phonology. and the 2pl ending -*tha*, but, in addition to those, also another ending -*tu* (-*du*). More than twenty years ago, Jamison (2000: 78, fn. 49) wrote that this ending -*tu* (-*du*) “deserves another look”, which is what this paper aims to do. As part of the argumentation, the pronoun *tusya*/*tus̱a* ‘you’ needs to be scrutinized as well.

The aims of this paper are twofold. First, I will argue that *tusya*/*tus̱a* is not a genitive singular (gen.sg), as per Burrow (1937: 32–33), but the direct plural (dir.pl)2I use the term direct case (dir) for the merger of the nominative (nom) and accusative (acc) case in Niya Prakrit, thereby following the conventions of Baums and Glass (2002–). of the second person pronoun. Based on this new analysis, I will also provide a new etymology of *tusya*/*tus̱a*. Second, I hope to demonstrate that -*tu* (-*du*) is not a 2sg ending (so e.g. Burrow 1937: 43; Jamison 2000: 78, fn. 49), but a 2pl ending, thereby replacing the older ending -*tha*. I will present arguments in favour of this supposition from (1) a quantitative comparison of -*tu* (-*du*) with -*si* (-*s̱i*) and -*tha* from the perspective of “Tense, Aspect and Mood (TAM)”, (2) a study of the pronouns with which these second person endings correlate and (3) an investigation of the grammatical number of these second person verb forms.

In order to properly understand this last point on grammatical number, a few words ought to be said on the pragmatic context of the Niya documents. With the exception of a few literary pieces (e.g. CKD 510),3My work is heavily indebted to the online Gāndhārī corpus in Baums and Glass (2002–), whose reference system I follow here. CKD stands for “Corpus of Kharoṣṭhī Documents” and CKI for “Corpus of Kharoṣṭhī Inscriptions”. all Niya documents are “administrative documents”, but there is still considerable variation among them, as the corpus consists of such different types of texts as contracts, lists related to taxation, letters, royal commands and decrees etc.4The terminology has been adopted from Høisæter (2020: 106–110; cf. also Padwa 2007: 105–117). While for “royal commands” and “royal decrees” the king is the sender and some official(s) the addressee(s), “letters” are usually sent between different officials. The main difference between “royal commands” and “royal decrees” is that the latter are usually more detailed and written on leather rather than on wood. It should not be surprising that second person pronouns and verb forms are mainly found in letters and in royal commands and decrees. For these types of texts, it is usually clear how many people are addressed, given that the addressees are listed in the formulaic introduction to these texts and in the delivery instructions. On this basis, one can infer whether a certain verb form or pronoun refers to one person or to more and should thus be classified as singular or plural. Therefore, these addressees will play an important part in the argumentation of this paper.

In what follows, Niya Prakrit is considered as a language in its own right, different from the Gāndhārī of the inscriptions and the literary texts. This is because the verbal ending -*tu* (-*du*) is only used as an imperative in these variants of Gāndhārī (cf. e.g. *bhavatu* and the like ‘may it be’ in many *ex voto* inscriptions, e.g. CKI 145; 147 etc.) and because the pronoun *tusya*/*tus̱a* is entirely lacking in such texts. On a more general level, the detailed analysis of the verbal ending -*tu* (-*du*) and the pronoun *tusya*/*tus̱a* should once again highlight “that the language of the Documents [i.e. Niya Prakrit] is both too systematic and too rich to allow it to be regarded as an artificial hybrid jargon” (Jamison 2000: 64, fn. 7, *contra* Fussman 1989: 440).5The closely related Kuča Prakrit (Ching 2013; Schmidt 2001) and Khotan Prakrit (CKD 661) will only be tangentially touched upon here through their lack of attestations of second person verb forms.


This paper is structured as follows. In Section 2, I question Burrow’s analysis of *tusya*/*tus̱a* as a gen.sg (Section 2.1), I provide evidence that *tusya*/*tus̱a* is in fact dir.pl (Section 2.2) and I will suggest a new etymology for this form (Section 2.3). In Section 3, I first offer a brief overview of the verbal system of Niya Prakrit as it is understood in earlier scholarship (Section 3.1), I then discuss a few aspects in which the ending -*tu* (-*du*) differs from -*si* (-*s̱i*) and -*tha* with regard to TAM (Section 3.2), I further argue that -*tu* (-*du*) should be classified as 2pl (Section 3.3) and I finally present a few diachronic notes (Section 3.4). The concluding Section 4 summarizes the main results.

## 2 The second person in the pronominal system of Niya Prakrit

### 2.1 The pronominal system of Niya Prakrit

Table 1 summarizes Burrow’s (1937: 32–33) analysis of the pronominal system of Niya Prakrit. The third person pronouns are not included, because Burrow (1937: 33–35) treats them as demonstrative pronouns. An analysis of these third person pronouns does in any case not contribute anything essential to the argumentation of this paper. The reader should take the following points into account. As in the rest of this paper, I use the term “direct case” for the merger of the nominative and the accusative case. Etymologically, these pronouns are all derived from the OIA nominatives. Infrequent variant spellings of the default forms are put in between brackets. Similar to what has happened in other MIA languages, the genitive and dative case have merged in Niya Prakrit. As far as the pronouns are concerned, both etymological datives and etymological genitives are found. While synchronically speaking they belong together, these are still separated from one another in the table, the old datives being listed in the first row and the old genitives in the second row.

**Table 1:** Niya Prakrit pronouns according to Burrow.

Case		1sg	2sg	1pl	2pl
dir		*ahu* (*ahaṃ*/*ahum*)	*tuo* (*tuvo*/*tu*)	*veya(ṃ)* (*vayaṃ*)	
gen/dat	< dat	*mahi*	*tahi* (*tehi*/*dahi*)	*amahu*/*asmahu* (*amaho*)	*tumahu*/*tusmahu* (*tumaho*)
	< gen	*mama*	*tava*/*to(ṃ)mi* *tusya* (*tus̱a*)	*asmag̱a*/*asmakaṃ*	*tusmag̱a*/*tusmakaṃ*
ins		*maya*		*asmabhi* *asmehi* (used as a gen) *asmag̱ena*	
rest forms					*yuṣme* (Sanskritic acc.pl, used with *agrata* ‘in front of’)

Various aspects of the Niya Prakrit pronouns are in need of further investigation. It remains, for instance, to be studied whether *to(ṃ)mi* is a mere variant of *tava* (Burrow 1937: 96), or whether this form should be analysed differently. Another topic for further research is the synchronic distinction between the old datives 1sg *mahi*; 2sg *tahi* (*tehi*/*dahi*); 1pl *amahu*/*asmahu* (*amaho*); 2pl *tumahu*/*tusmahu* (*tumaho*) and the old genitives 1sg *mama*; 2sg *tava*/*to(ṃ)mi*; 1pl *asmag̱a*/*asmakaṃ*; 2pl *tusmag̱a*/*tusmakaṃ*. As the etymological datives are more frequent than the etymological genitives,6I counted 119 tokens of *mahi* in 103 docs. versus 4 tokens of *mama* in 4 docs. and 100 tokens of *tahi* (*tehi*/*dahi*) in 62 docs. versus 5 tokens of *tava* in 1 doc. and 23 tokens of *to(ṃ)mi* in 23 docs. Regarding the plural forms, I have found 25 tokens of *amahu*/*asmahu* (*amaho*) in 22 docs. versus 10 tokens of *asmag̱a*/*asmakaṃ* in 6 docs. (most of which are in Kuča Prakrit) and 35 tokens of *tumahu*/*tusmahu* (*tumaho*) in 26 docs. versus 4 tokens of *tusmag̱a*/*tusmakaṃ* in 4 docs. it appears that the former are the productive forms, while the latter appear to be Sanskritic archaisms, possibly sociolinguistically marked for register. This impression needs, however, further research.

The point I want to focus on here is that Burrow’s system surprisingly lacks a dir.pl for the second person pronoun. In other words, why does Niya Prakrit lack a second person functional counterpart of first person *veya(ṃ)* (*vayaṃ*) ‘we.dir.pl’? While it is in theory possible that such a form has simply not yet turned up in our corpus, it is equally possible that the dir.pl of the second person pronoun is hidden in plain sight. In fact, when looking at Table 1, it catches the eye that Burrow assumes *tusya* (*tus̱a*) to be an additional form of the gen/dat.sg of the second person. Yet, this *tusya* (*tus̱a*) lacks then a counterpart in the first person, where there is only *mahi*, corresponding to *tahi* (*tehi*/*dahi*), and *mama*, corresponding to *tava*/*to(ṃ)mi*. It, therefore, seems worthwhile to investigate whether this *tusya* (*tus̱a*) could not be the missing dir.pl of the second person. In Section 2.2, I argue this is indeed the case.

### 2.2 *tusya* (*tus̱a*) is dir.pl

In this subsection, I provide evidence that *tusya* (*tus̱a*) should be classified as a dir.pl rather than as a gen.sg. In Table 2, I list all the securely attested examples of *tusya* (*tus̱a*), thereby indicating how many people are addressed and how *tusya* (*tus̱a*) is used syntactically. As briefly discussed in Section 1, Niya Prakrit second person pronouns (and verbal forms) are mainly used in letters and decrees. On the basis of the formulaic introduction to these types of texts, one can infer to how many people the letter is addressed and, by implication, thus also whether a pronoun as *tusya* (*tus̱a*) is used to refer to a plurality of persons or to only one person. Besides, the syntactic usage of *tusya* (*tus̱a*) will help us to properly define the case form of this pronoun.

**Table 2:** The use of *tusya (tus̱a)* in Niya Prakrit.

CKD	Form	Number of people addressed	Syntactic usage
106	*tusya*	2	Agent of *saṃghaṭidavo* ‘to be pieced together’
119	*tus̱a*	2	Subject of visarjiṣ(*ya)tu ‘you will send’
140	*tusya-tra* *tusya*	2	– poss.gen with *divyaśariraaroga* ‘health of the divine body’ – Indirect object of *prahidemi* ‘I sent’
157	*tusya*	3	Subject of *baṃnides̱i* ‘you tied up’
159	*tusya*	2	Dependent on *paride* ‘from’
247	*tusya*	2	Subject of arog(*etu) ‘you are healthy’ (cf. Section 3.3 for this restoration).
272	*tusya*	1	Subject of *asidetha* ‘you settled’
278	*tus̱a*	2	Subject of *[s̱a]ti hutu* ‘you should be on your guard’
320	*tus̱a*	2	Subject of *ukastetu* ‘you went away’
470	*tusya*	2	Subject of *katvetha* [*recte: kaṭetha*] ‘you did’
475	*tusya*	2	poss.gen with *arog̱a* ‘health’
519	*tusya* *tusya*	2	– Indirect object of inf *kartu* ‘to do’ – poss.gen with *pitu* ‘father’
562	*tusya*	1	Subject of *karetu* ‘you do’
578	*tusya*	2	poss.gen with *niryig̱a* ‘relaxation’ (?)
639	*tusya*	1	Subject of *ṣayatu* ‘you take hold of’
690	*tusya*	at least 2 (cf. gen.pl *priyadarśanana* in the introduction)	poss.gen with *arog̱a* ‘health’
714	*tus̱a* *tus̱a* *tus̱a*	2	– Subject of *arog̱etu* ‘you are healthy’ – Subject of *karetu* ‘you do’ – Subject of *achiṃnidetu* ‘you cut off’
794	*tusya*	1	Subject of *janatu* ‘you (should) know’
796	*tusya*	4	Subject of *janatu* ‘you (should) know’

On the basis of Table 2, one can make the following observations. First of all, out of the 19 documents that unmistakably contain a form of *tusya* (*tus̱a*), 15 mention more than one person in the opening to the letter (∼79%). Counting per token, 20 out of the 23 instances of *tusya* (*tus̱a*) are found in a document that is addressed to more than one person (∼87%). These statistics very much suggest that *tusya* (*tus̱a*) should be regarded a plural pronoun. Moreover, in the few cases *tusya* (*tus̱a*) occurs in a letter which addresses only one person (i.e. in CKD 272; 562; 639; 794), the pronoun is consistently used as the subject of a verb ending in -*tha* or -*tu*, which, as argued in Section 3.3, both appear to be used for 2pl. As regards the syntax of *tusya* (*tus̱a*), 14 out of 23 tokens of *tusya* (*tus̱a*) are used in subject function. This observation is a further confirmation that *tusya* (*tus̱a*) is the functional counterpart of 1pl *veya(ṃ), vayaṃ* ‘we’ which was missing in Burrow’s interpretation of the pronominal system. The fact that *tusya* (*tus̱a*) is also found in other grammatical functions can be explained on the basis of the general case confusion in Niya Prakrit (see Burrow 1937: 22–30, but also Jamison 2000). In short, the Niya Prakrit personal pronouns of the first and second person should rather be understood as in Table 3, whereby I indicate the main difference with Burrow’s analysis in bold face.

**Table 3:** Niya Prakrit pronouns according to this study.

Case		1sg	2sg	1pl	2pl
dir		*ahu* (*ahaṃ*/*ahum*)	*tuo* (*tuvo*/*tu*)	*veya(ṃ)* (*vayaṃ*)	***tusya*** **(*tus̱a*)**
gen/dat	< dat	*mahi*	*tahi* (*tehi*/*dahi*)	*amahu*/*asmahu* (*amaho*)	*tumahu*/*tusmahu* (*tumaho*)
	< gen	*mama*	*tava*/*to(ṃ)mi*	*asmag̱a*/*asmakaṃ*	*tusmag̱a*/*tusmakaṃ*
ins		*maya*		*asmabhi* *asmehi* (used as a gen) *asmag̱ena*	
rest forms					*yuṣme* (Sanskritic acc.pl, used with *agrata* ‘in front of’)

### 2.3 A new etymology for *tusya*/*tus̱a*

Now that it is clear that *tusya* (*tus̱a*) should be analysed as a dir.pl, a new etymology for this pronoun presents itself. Based on his interpretation of *tusya* (*tus̱a*) as a gen.sg, Burrow (1937: 32–33) segmented *tusya* (*tus̱a*) as the pronominal stem *tu*- and the nominal gen.sg ending -*sya*. While this etymology would be plausible if *tusya* (*tus̱a*) were indeed gen.sg, it is much less so when *tusya* (*tus̱a*) is in fact dir.pl, because the development of a 2sg to a 2pl pronoun is typologically very rare (Helmbrecht 2015: 184–188). In addition, the other direct case forms, i.e. *ahu* (*ahaṃ*/*ahum*), *tuo* (*tuvo*/*tu*) and *veya(ṃ)* (*vayaṃ*), are all etymological nominatives. Therefore, it is more likely that *tusya* (*tus̱a*) should be derived from OIA *yūyam* ‘you.nom.pl’ with analogical replacement of *yū*- by the oblique stem *tus*- seen in Niya *tusmahu* ‘you.gen/dat.pl’ and *tusmag̱a*/*tusmakaṃ* ‘you.gen/dat.pl’.7For this type of analogical replacement in MIA second person pronouns, see Oberlies (2019 [2001]: 267–270) on Pāli and von Hinüber (2001 [1986]: 253–254) on other Prakrits. If we want to follow Insler (1988–1990 [1991]), this type of analogy may even have occurred already in R̥gvedic Sanskrit. In this article, Insler has argued that *tū́yam*, commonly interpreted as an adverb meaning ‘quickly’, should in fact be analysed as a conflation of 2sg *tū́* ‘you’ and 2pl *yūyám* ‘you’. The loss of the final -*m* in *tusyam (← *yūyam*) is paralleled by *veya* < *vayam* ‘we.nom.pl’ and e.g. the dir.sg ending of the *a*-stems, i.e. -*a* < -*am* (see Burrow 1937: 22).

## 3 The second person in the verbal system of Niya Prakrit

### 3.1 The verbal system of Niya Prakrit

As is the case with other MIA languages, Niya Prakrit has simplified its verbal system as compared to OIA. According to the *communis opinio* (see Burrow 1937: 43–56), Niya Prakrit has eight moods (indicative; imperative; causative; optative; infinitive; absolutive; gerundive; participle), three tenses (present; past; future), three persons (1st; 2nd; 3rd) and two numbers (sg; pl). Traces of the OIA middle voice are rare and likely artificial archaisms; the passive, with suffix -*ya*-, is also used only occasionally. There are only two productive present stem formations, one with a stem in -*a* and one with a stem in -*e* (< -*aya*-). Taking the verb *denati* ‘to give’ as an example, Table 4 gives an overview of how Burrow seems to understand the present conjugation in Niya Prakrit (not all the forms of *denati* given are actually attested in the texts). The reader should note that Burrow assumes that the ending -*tu* (-*du*) alternates with the 2sg ending -*si* (-*s̱i*).

**Table 4:** Niya Prakrit present tense according to Burrow.

1sg	*denami* ‘I give’
2sg	*denasi* (*denas̱i*); *denatu* (*denadu*) ‘you (sg) give’
3sg	*denati* (*denadi*) ‘he/she gives’
1pl	*denama* ‘we give’
2pl	*denatha* ‘you (pl) give’
3pl	*denaṃti* ‘they give’

The same endings as for the present tense are used (1) for the future tense, where the suffix -*iśa*- (-*iṣya*-) is added to the present stem, (2) for the causative mood, which is characterised by the suffix -*ave*- < -*āpaya*-, and (3) for the optative mood (only used for present tense), which uses the suffix -*eya*-. In the past tense, the stem of the verb derives from the OIA verbal adjective in -*ta* (see Barchi and Peschl forthcoming; Burrow 1937: 50–53), to which enclitic forms of ‘to be’ are added. Again using the verb *denati* ‘to give’ as an example, the (partially reconstructed) paradigm of the past tense as it is understood by Burrow is presented in Table 5. Here again, Burrow assumes that the ending -*tu* (-*du*) is another 2sg ending, next to -*si* (-*s̱i*).

**Table 5:** Niya Prakrit past tense according to Burrow.

1sg	*ditemi* ‘I gave’
2sg	*ditesi* (*dites̱i*); *ditetu* (*ditedu*) ‘you (sg) gave’
3sg	*dita* ‘he/she gave’
1pl	*ditama* ‘we gave’
2pl	*ditetha* ‘you (pl) gave’
3pl	*ditaṃti* ‘they gave’

In what follows, I will re-examine Burrow’s suggestion that the verbal ending -*tu* (-*du*) is an alternative to the ending -*si* (-*s̱i*), coming to the conclusion that the ending -*tu* (-*du*) is, *pace* Burrow, not a 2sg ending, but is in fact replacing the 2pl ending -*tha*. The quantitative comparison between -*tu* (-*du*), -*si* (-*s̱i*) and -*tha* undertaken in the next subsection (Section 3.2) will yield some observations that already point in this direction. Further arguments are later on given in Section 3.3.

### 3.2 TAM of the endings -*si* (-*s̱i*); -*tu* (-*du*); -*tha*

In the present subsection, the “Tense, Aspect & Mood (TAM)” of the Niya second person verbal endings, i.e. -*si* (-*s̱i*), -*tu* (-*du*) and -*tha*, are subjected to a quantitative analysis, in order to understand how these endings fit within the Niya Prakrit verbal morphology. Because of the focus on morphology, “tense” and “mood” will be our main concern, while the syntactic usage of the endings, here loosely captured under the term “aspect”, will only be briefly touched upon.8It remains to be investigated in detail which aspectual readings are possible for the different tenses in Niya Prakrit, the results of which could then be compared to Hoose’s (2020) conclusions on the aspectual usage of the past tenses in Pāli and Jaina-Mahārāṣṭrī.


Given that the inherited 2pl ending -*tha* does not require much comment, I will discuss this ending first. -*tha* is found only very infrequently (only 6 types/8 tokens),9
*achiṃnidetha* ‘you have cut off’ (CKD 275); *asidetha* ‘you have settled’ (CKD 272); *ichidetha* ‘you have wished’ (CKD 705); *kiṭatha* (*katvetha* to be corrected into *kaṭetha*, cf. Burrow 1937: 81) ‘you have made’ (CKD 213; 470 (2×)); *picavidetha* ‘you have handed over’ (CKD 375); *vis̱arjidetha* ‘you have sent’ (CKD 162). but, what is more, -*tha* is also only used in past tense forms. While not previously observed, these statistics are suggestive of a low productivity of the ending -*tha.* One, moreover, wonders how 2pl was then expressed in present and future, as the ending -*tha* is not found in these tenses.

As the basis for a comparison between the endings -*si* (-*s̱i*) and -*tu* (-*du*), Tables 6 and 7 give an overview of how often these endings are used in the present, past and future tense and in the optative mood.

**Table 6:** A quantitative study of the TAM of the second person ending *-si (-s̱i)*.

Tense/mood	Type frequency	Type percentage	Token frequency	Token percentage
Present tense^a^	23	38	45	38
Past tense^b^	22	37	50	43
Future tense^c^	11	18	18	15
Optative mood^d^	4	7	4	3
**TOTAL**	**60**	**100**	**117**	**100**

^a^ *achinas̱i* ‘you cut off’ (CKD 211; 450); *aprochas̱i* ‘you do not ask’ (CKD 819); *arog̱e[si] (arog̱es̱i)* ‘you are healthy’ (CKD 302; 305; 385; 666; 721; 797; 840); *avajas̱i* ‘you apply’ (CKD 448); *ichasi* ‘you wish’ (CKD 317); *oḍ̱esi* ‘you let go’ (CKD 317); *kares̱i* (*karesi*/*kurvasi*) ‘you make’ (CKD 7; 46; 144 (2×); 538; 719; 775; 797); *karmavisi* ‘you put to work’ (CKD 313); *khaṃnavaṭag̱esi (kanavaṭes̱i)* ‘you play the procrastinator (?)’ (CKD 358; 634); *garahasi* ‘you complain’ (CKD 538); *janes̱i* (*jaṃnasi*/*jaṃnas̱i*/*janasi)* ‘you know’ (CKD 106; 140 (3×); 305); *denasi* ‘you give’ (CKD 358); *nikhales̱i* ‘you take out (?)’ (CKD 211); *picavesi* ‘you hand over’ (CKD 553); *bhavas̱i* ‘you are’ (CKD 376); *mav̱esi* ‘you declare (?)’ (CKD 538); *mahatvas̱i* ‘you are an official’ (CKD 211); *lihas̱i* ‘you write’ (CKD 317); *viṃñaves̱i (viṃñav̱es̱i)* ‘you let know’ (CKD 283; 292; 358; 387); *vithav̱esi* ‘you hold back’ (CKD 639); *vis̱ajesi* ‘you send’ (CKD 526); *saṃdhiśas̱i* ‘you inform’ (CKD 819); *si* ‘you are’ (CKD 113). ^b^ *anavides̱i* ‘you have given instructions’ (CKD 83); *anides̱i* ‘you have brought’ (CKD 547); *kiḍ̱esi* (*krides̱i/kiṭas̱i*) ‘you have made’ (CKD 46; 107; 387; 775); *gades̱i* ‘you have gone’ (CKD 106); *gameṣides̱i* ‘you have sought’ (CKD 361); *giḍ̱esi* (*ginides̱i*) ‘you have taken’ (CKD 63 (2×); 144; 414); *thavides̱i* (*thavites̱i*) ‘you have fixed’ (CKD 201; 376; 797); *ditesi* ‘you have given’ (CKD 223; 624); *nikhalites̱i* ‘you have taken out (?)’ (CKD 376); *parimargides̱i* ‘you have investigated’ (CKD 578); *picavides̱i* ‘you have handed over’ (CKD 552); *prahides̱i* (*prahites̱i/prahidesi/prehides̱i*) ‘you have sent’ (CKD 106; 128; 206; 211; 272; 283; 357; 358; 361; 376; 387; 819); preṣi[t](*e)(*s̱i) ‘you have sent’ (CKD 414); *baṃnides̱i* ‘you have bound’ (CKD 157); *vaj̱ites̱i* ‘you have read’ (CKD 376); *vikrides̱i* ‘you have sold’ (CKD 106); *vithavidesi* ‘you have held back’ (CKD 206); *vibhaśites̱i* ‘you have decided’ (CKD 625); *vis̱arjides̱i* (*vis̱ajides̱i*) ‘you have sent’ (CKD 69; 86; 133; 160; 211; 309); *sajavides̱i* ‘you have made ready’ (CKD 376); *saṃtiṭhes̱i* ‘you have informed’ (CKD 83; 663); *hudesi* (*hudes̱i*) ‘you have been’ (CKD 309; 546; 625). ^c^ *agamiṣyas̱i* ‘you will come’ (CKD 211; 722); *kariṣyasi* (*kariṣyas̱i*/*kariśas̱i*) ‘you will make’ (CKD 83; 161 (2×); 387; 635); *giṃniṣyas̱i* ‘you will take’ (CKD 448); *choriṣyasi* ‘you will steal’ (CKD 385); *dasyasi* ‘you will give’ (CKD 358); *paribujiśasi* (*bujiśasi*) ‘you will understand’ (CKD 356; 433); *preṣeyiṣyasi* ‘you will send’ (CKD 399); *vithiṣyasi* (*vitha[viṣya]s̱i*) ‘you will hold back’ (CKD 83; 376); *[v]is̱ajiśasi* ‘you will send’ (CKD 217); *vyoṣiśas̱i* ‘you will reimburse’ (CKD 165); *śodheyiṣyas̱i* ‘you will pay’ (CKD 635). ^d^ *kareyas̱i* ‘you may make’ (CKD 373); *praśameyas̱i* ‘you may calm down’ (CKD 373); *preṣeyaṃs̱i* ‘you may send’ (CKD 399); *vis̱arjeyasi* ‘you may send’ (CKD 696).

**Table 7:** A quantitative study of the TAM of the second person ending *-tu (-du)*.

Tense/mood	Type frequency	Type percentage	Token frequency	Token percentage
Present tense^a^	19	47	69	37
Past tense^b^	6	15	7	4
Future tense^c^	15	38	108	59
Optative mood	0	0	0	0
**TOTAL**	**40**	**100**	**184**	**100**

^a^ *arog̱etu* ‘you are healthy’ (CKD 399; 714; 819); *ichatu* ‘you wish’ (CKD 86); *upajivatu* ‘one should live in dependence of’ (CKD 31 + 764); *karitu (kareṃtu*/*karetu*) ‘you (should) do’ (CKD 45; 177; 373; 399 (3×); 423; 438; 562 (3×); 714); *gachaṃtu* ‘you go’ (CKD 373); *choretu* ‘you should abandon’ (CKD 134); *janatu* ‘you (should) know’ (CKD 794; 796); *darśavetu* ‘you show’ (CKD 761); *davyatu* ‘it should be given’ (cf. Burrow 1937: 45) (CKD 399); *denatu* ‘you (should) give’ (CKD 135; 475); *picavetu* ‘you hand over’ (CKD 439); *prasavetu* ‘you should produce’ (CKD 338); *pruchitu* ‘you should ask’ (CKD 295); *margetu* ‘you search’ (CKD 399 (2×)); *vacitu* ‘you should read’ (CKD 399; less likely an absolutive as Burrow [1940: 81] takes it, as Niya Prakrit absolutives normally end in -*ti*); *viṃñav̱etu* (*viṃñav*⟨*e⟩*tu*) ‘you inform’ (CKD 292 (2×); 357; 358); *vis̱ajetu* ‘you send’ (CKD 247; 357); *ṣayatu* ‘you take hold of’ (CKD 639); *hotu (hodu*/*hutu*/*hoṃtu/bhavetu)* ‘it should be’ (CKD 31 + 764; 39; 68; 100; 152; 217; 244; 248; 272 (4×); 278; 307; 317; 320; 329; 341; 358 (2×); 362; 367; 370; 399; 585 (2×); 633; 634; 721; 722; 815). For the adopted translations, see below. ^b^ *achiṃnidetu* ‘you have cut off’ (CKD 714); *anavidetu* ‘you have given instructions’ (CKD 162); *ukastetu* ‘you have gone away’ (CKD 320); *lihitetu* ‘you have written’ (CKD 157); *vithitetu* ‘you have held back’ (CKD 519); *vis̱arjidetu* (*vis̱arjitetu*) ‘you have sent’ (CKD 126; 399). ^c^ *agaṃmiṣyatu* (*agachiśatu*) ‘you will come’ (CKD 399; 634); *aniṣyatu* ‘you will bring’ (CKD 517; 554); *oḍ̱iṣyatu* (*oḍ̱iśaṃtu*) ‘you will let go’ (CKD 125; 157; 159; 320; 546; 633); *kariṣyatu* (*kāriṣyatu*) ‘you will do’ (CKD 68; 106; 206; 320; 367; 450; 634; 819); *dāsyatu* ‘you will give’ (CKD 367); *nivartiṣ[ya]tu* ‘you will return’ (CKD 634); *paḍ̱ichiṣyatu* ‘you will receive’ (CKD 517); *paribujiśatu* (*bujiśatu*/*paribhujiśatu*/*paribujiśadu*) ‘you will understand’ (CKD 1; 3; 6; 7; 9; 11; 12; 15; 18; 20; 21; 24; 27; 29; 32; 33; 36; 37; 39; 45; 47; 49; 53; 61; 62; 71; 124 (2×); 192; 212; 223; 235; 240; 262; 265; 286; 297; 308; 312; 344; 364; 386; 393; 408; 423; 473; 479; 480; 481; 482; 484; 492; 503; 509; 526; 528; 530; 538; 542 (2×); 545; 548; 551; 555; 606; 636; 719; 720; 729; 734 (2×); 736; 738; 741); *labhiśaṃtu* (*labhiśatu*) ‘you will take’ (CKD 633 (2×); 635); *vikriśaṃtu* ‘you will sell’ (CKD 633); *vithiṣyatu* ‘you will hold back’ (CKD 165); *vis̱ajiṣyatu* (visarjiṣ(*ya)tu) ‘you will send’ (CKD 68; 119; 165; 714); *vyoṣiśatu* ‘you will reimburse’ (CKD 714); *sarajiśatu* ‘you will agree’ (CKD 399); *hakṣ̄atu* (*akṣ̄atu*) ‘you will be’ (CKD 4; 83).

The most intriguing conclusion one can draw from Tables 6 and 7 is that the ending *tu* (-*du*) is much less frequently used in past tense forms than the ending -*si* (-*s̱i*). This difference can be most clearly noticed if one looks at the token frequency, as there are only 7 instances of a past tense ending in -*tu* (-*du*) (∼4%) as against 50 instances of a past tense in -*si* (-*s̱i*) (∼43%). This rarity of past tense forms in -*tu* (-*du*) seems to indicate that past forms in -*tu* (-*du*) are less firmly anchored within the overall verbal system of Niya Prakrit. One is moreover inclined to think that this infrequency of the ending -*tu* (-*du*) in past tense forms is connected to the fact that the ending -*tha* is exclusively found in the past tense. In view of this, it would seem quite possible that -*tu* (-*du*) has taken over the place of -*tha* in present and future tense and is, by the time of our evidence, also gradually spreading towards the past tense. I will build further on this hypothesis in Section 3.3.

Tables 6 and 7 allow for a few other observations. Though slightly less important, these seem to be further confirmation that the endings -*si* (-*s̱i*) and -*tu* (-*du*) are, *pace* Burrow, not mere variants of one another. Future tense forms are, for instance, more frequent with the ending -*tu* (-*du*) (type frequency 37%; token frequency 59%) than with -*si* (-*s̱i*) (type frequency 18%; token frequency 15%). One should note, however, that, as far as token frequency is concerned, the data are slightly skewed because of the high frequency (74 tokens) of the verb form *paribujiśatu* (*bujiśatu/paribhujiśatu/paribujiśadu*) ‘you will understand’, which occurs in a formulaic sentence one encounters time and again in the Niya documents. Additionally, it appears that the ending -*tu* (-*du*) is never used for optatives, while there are a few instances (4 types; 4 tokens) of optatives ending in -*si* (-*s̱i*).10The lack of optatives with the ending -*tu* (-*du*) was implicitly noted already by Burrow (1937: 46–47), as he only gives -*si* (-*s̱i*) as an optative ending for the second person.


On top of these statistic differences between -*tu* (-*du*) and -*si* (-*s̱i*), there is one more difference in their syntactic usage that should not go unremarked, i.e. the ending -*tu* (-*du*) can be used to express commands in the present tense, which is not possible with -*si* (-*s̱i*). In his Niya Prakrit grammar (1937: 45), Burrow only acknowledges *hotu* (*hodu*/*hutu*/*hoṃtu*/*bhavetu*) ‘it should be’ (cf. also Balbir 1990: 26) and *davyatu* ‘it should be given’ (CKD 399) as old imperative forms.11According to Burrow, gerundives have largely replaced the old imperatives, but gerundives seem to be suited better for the administrative, formalized jargon of the Niya documents than more assertive second person imperatives (similarly Caillat 1990: 20; cf. also Luukka and Markkanen 1997 on impersonal constructions as a “hedging” device). This sociolinguistic explanation also fits well with the few second person imperatives that are actually attested in the Niya documents. In CKD 27, *dehi* ‘give’ is used in a quote of direct speech and in CKD 617 *śrunahi* ‘listen’ is attested in a poetic piece in so-called “Gāndhārī Hybrid Sanskrit” (Salomon 2001). An anonymous reviewer also refers to Levman’s (2020: 169–193) argument that the usage of impersonal constructions in Buddhist teachings could be seen as a linguistic expression of the Buddha’s concept of *anattā*, i.e. the idea there is no human soul, and wonders whether the same concept could also have influenced the administrative writing style of the Buddhist Shanshan kingdom. Yet, a more attractive comparison can be drawn with the predilection for impersonal constructions in Imperial Aramaic letters which is noted by Gzella (2021: 31). While more systematic research on the “Aramaic substrate” in the Niya documents remains a *desideratum*, it has been noted before (e.g. Sims-Williams 1996: 81; Yakubovich 2006: 338) that parts of the phraseology of the Niya documents go back to the Imperial Aramaic writing tradition of Achaemenid times. Yet, as Burrow seems to have recognized by the time of his translation of the Niya documents (1940), there are a few more present tense forms ending in -*tu* (-*du*) that are synchronically still used to express a command in the Niya documents. As will be discussed in somewhat more detail in Section 3.4, this synchronic fact suggests a historical connection with the OIA third person imperative ending in -*tu*. What follows is a list of the present forms in -*tu* (-*du*) that seem to express commands in the presently available Niya Prakrit corpus: CKD 31 + 764 *upajivatu* ‘one should live in dependence of’; CKD 134 *choretu* ‘you should abandon’; CKD 177 [*karitu*] ‘you should make’; CKD 295 *pruchitu* ‘you should ask’; CKD 338 *prasavetu* ‘you should produce’; CKD 373 *kareṃtu* ‘you should make’; CKD 399 *vacitu* ‘you should read’; CKD 475 *denatu* ‘you should give’; CKD 794 *janatu* ‘you should know’. A brief look at the negations used with present forms in -*tu* (-*du*) confirms that this ending -*tu* (-*du*) can be used in two distinct functions in the present tense, i.e. for declarative statements and for commands.12More generally, Jamison’s (2000: 78, fn. 49) impression that negations are more often found with verbs ending in -*tu* (-*du*) than with those ending in -*si* (-*s̱i*) seems to be correct. I have counted 25 tokens of negated verbs ending in -*si* (-*s̱i*) (out of a total of 117 verb forms in -*si* (-*s̱i*) ∼ 21%) versus 104 tokens of negated verbs ending in -*tu* (-*du*) (out of a total of 184 verb forms in -*tu* (-*du*) ∼ 56.5%). Yet, with regard to the forms in -*tu* (-*du*), it should be noted that 74 tokens concern the same type *paribujiśatu* (*bujiśatu/paribhujiśatu/paribujiśadu*) ‘you will understand’, which, as noted above, occurs in a formulaic sequence in Niya Prakrit. As the high frequency of this verb skews the statistics, I have also made a calculation of what happens with the percentage when these forms are simply left out completely. In that case, there are 30 negated verb forms in -*tu* (-*du*) out of a total of 110, which comes to 27%. This means that verb forms in -*tu* (-*du*) (27%) are still more often negated than those in -*si* (-*s̱i*) (21%), but the distinction seems too insignificant to build conclusions on. Similar notes can be made on Jamison’s suspicion that verbs ending in -*tu* (-*du*) are more often found in subordinate clauses than those in -*si* (-*s̱i*). Of the latter type, I have counted 29 tokens (out of a total of 117 verb forms in *si* (-*s̱i*) ∼ 25%), while I found 105 tokens of -*tu* (-*du*) in subordinate clauses (out of a total of 184 verb forms in -*tu* (-*du*) ∼ 57%). Yet, these statistics are once again biased by the 74 tokens of *paribujiśatu* (*bujiśatu/paribhujiśatu/paribujiśadu*) ‘you will understand’. When these are not counted, there are 31 examples of verbs in -*tu* (-*du*) in subordinate clauses out of a total of 110, which brings us to 28%. This percentage is still slightly higher than for verbs in -*si* (-*s̱i*) (25%), but again not particularly noteworthy. Verbs ending in -*tu* (-*du*) use the negation *na* ‘not’ in declarative statements,13CKD 45 *na karitu* ‘you do not make’; CKD 86 *na ichatu* ‘you do not wish’; CKD 247 *na … vis̱ajetu* ‘you do not send’; CKD 399 (2×) *na … karetu* ‘you do not make’; CKD 562 *na … karetu* ‘you do not make’; CKD 796 *na janatu* ‘you do not know’. while *ma iṃ ci* ‘not (at all)’ < OIA *mā kiṃ cit* is the default negation when present forms in -*tu* (-*du*) express a command or, more precisely in this case, a prohibition.14CKD 134 *ma iṃ ci … choretu* ‘you should not abandon’; CKD 295 *ma iṃ ci pruchitu* ‘you should not ask’; CKD 399 *ma iṃ ci … davyatu* ‘it should not be given’. Needless to say, this distinction is in line with OIA (Delbrück 1888: 541–546; Speijer 1886: 315–320).

In short, the syntactic usage of verbs containing the endings -*si* (-*s̱i*) and -*tu* (-*du*) and especially the frequency of these endings in specific tenses makes it clear that one cannot simply consider them variants of one another. It is, for instance, essential to take into account that the ending -*tu* (-*du*) is only infrequently used in past tense forms and that this same ending can be used in present tense forms to express commands.

### 3.3 -*tu* (-*du*) is 2pl

As one of the conclusions of the preceding subsection is that the 2pl ending -*tha* is only used for past tense forms, should there not be another 2pl ending for present and future tense? As -*tu* (-*du*) is not at all frequent in past tense forms, but more so in present and future tense, it seems worth investigating whether -*tu* (-*du*) is not rather the 2pl ending that is lacking for present and future (and which may then also be gradually taking ground in the past tense). The following two pieces of evidence seem in favour of this hypothesis. First, it will be pointed out that both the ending -*tu* (-*du*) and -*tha* correlate with the personal pronoun *tusya* (*tus̱a*) ‘you.dir.pl’, while the ending -*si* (-*s̱i*) is combined with *tuo* (*tuvo*/*tu*) ‘you.dir.sg’. Second, there is a general tendency for verbs ending in -*si* (-*s̱i*) to be attested in letters and decrees that are addressed to only one person, whereas verbs ending in -*tu* (-*du*) and -*tha* are by comparison found more often in documents that are sent to more than one person.15When these 2pl verb forms are found in letters that are sent to only one person, they can be explained as so-called “polite forms” (as with French *vous* ‘you.pl’). See below. These two arguments will be taken up in turn.

Regarding the first argument, it may first of all be noted that the ending -*tu* (-*du*) is less often combined with an overt personal pronoun in subject function than the other second person endings. Compare the statistics in Table 8.

**Table 8:** The amount of second person verb forms combined with an overt personal pronoun in subject function.

Verbal ending	Instances of this verbal ending	Instances of a combination between this verbal ending and a personal pronoun	%
-*si* (-*s̱i*)	117 tokens	37 tokens^a^	32
-*tu* (-*du*)	184 tokens	10 tokens^b^	5.4
-*tha*	8 tokens	2 tokens^c^	25

^a^See CKD 46; 63 (2×); 83; 106 (2×); 140 (3×); 144 (2×); 161; 165; 211 (2×); 302; 305; 309; 313; 317; 376 (2×); 385 (2×); 538; 546; 552; 625 (2×); 635; 666; 721; 722; 797 (2×); 840. In CKD 317, it is unclear whether *tuo* ‘you.dir.sg’ only modifies *ichasi* ‘you wish’ or also *oḍ̱esi* ‘you let go’ and I have counted this as only one token. ^b^See CKD 278; 320; 517; 562; 639; 714 (3×); 794; 796. ^c^See CKD 272; 470.

Related to this, verbs ending in -*tu* (-*du*) are never combined with both a personal pronoun in subject function and a nominal subject standing in apposition with the pronoun. For the verbal ending -*si* (-*s̱i*), on the other hand, there are five examples of this construction,16See CKD 63 (*tuo cozbo* ‘you, the *cozbo*’); 144 (*tuo cozbo Soṃjaka* ‘you, the *cozbo* Soṃjaka’); 165 (*tuo ṣoṭhaṃga Lýipeya* ‘you, the *ṣoṭhaṃga* Lýipeya’); 552 (*tuo bhaṭarag̱a* ‘you, the master’); 625 (*tuo cozbo Soṃjaka* ‘you, the *cozbo* Soṃjaka’). one of which (example sentence (1)) is found in CKD 144, where the king speaks to the *cozbo*-official Soṃjaka.

(1)

**Table:** 

*iśa*	***tuo***	***cozbo***	***Soṃjaka***	*a[si]yade*
here	you.dir.sg	*cozbo*.dir.sg	Soṃjaka.dir.sg	mouth.abl.sg
*anadi*		***giḍ̱esi.***			
command.dir.sg		receive.pst.2sg			
‘Here **you, the *cozbo* Soṃjaka, received** an oral command.’					
(Burrow 1940: 26)					

Second and still more important, when verbs ending in -*tu* (-*du*) are combined with an overt personal pronoun in subject function, this is, with only one exception, the pronoun *tusya* (*tus̱a*) ‘you.dir.pl’ (cf. Section 2.2). In this respect, -*tu* (-*du*) aligns with 2pl -*tha*, which also twice has *tusya* (*tus̱a*) as its subject, but not with 2sg -*si* (-*s̱i*), which is, notwithstanding one exception, by default found together with *tuo* (*tuvo*/*tu*) ‘you.dir.sg’. See Table 9 for the details. These observations are a strong indication that -*tu* (-*du*) should be regarded as 2pl and not as 2sg, as has been assumed before.

**Table 9:** The correlation between the pronouns *tuo (tuvo/tu)* ‘you.dir.sg’ and *tusya (tus̱a)* ‘you.dir.pl’ and the verbal endings *-si (-s̱i); -tu (-du); -tha.*

Verbal ending	*tuo* (*tuvo*/*tu*) as subject	*tusya* (*tus̱a*) as subject
-*si* (-*s̱i*)	36 tokens	1 token (*baṃnides̱i* ‘you have bound’ in CKD 157^a^)
-*tu* (-*du*)	1 token (*paḍ̱ichiṣyatu* ‘you will receive’ in CKD 517)	9 tokens
-*tha*	0 tokens	2 tokens

^a^ *baṃnides̱i* is in any case somewhat unexpected, as CKD 157 is addressed to more than one person.

Regarding the second argument that -*tu* (-*du*) is a 2pl ending, I have first counted in how many documents a particular second person ending is attested and I have then investigated whether these documents specify, either in the introduction or in the delivery instructions, to how many people this document was addressed. Having so defined my corpus, I then calculate the percentage of documents that are sent to only one person and those that are sent to more than one person.

If we apply the methodology described above to the verbal ending -*tha*, we obtain the following results. There are seven documents that contain verbs ending in -*tha,* one of which is too fragmentarily preserved to be of any use (CKD 705), which means there are six documents to be considered. Exactly half of those (3 docs. ∼ 50%) are addressed to only one person (CKD 213; 272; 275), while the other half (3 docs. ∼ 50%) is addressed to more than one person (CKD 162; 375; 470). While the latter examples do not necessitate further comment, the former ones can be accounted for if we assume that, precisely as in Sanskrit (cf. Speijer 1886: 195), the ending -*tha* could also be used as a polite form when referring to one person only. Needless to say, using a 2pl verb form as a polite form is typologically frequent (cf. e.g. French or Modern Greek).17The linguistic literature on this topic of polite forms, sometimes called “plurification”, is vast. See recently e.g. Heine and Song (2010: 129–134, 2011: 609) and Helmbrecht (2015: 181).


When doing the same calculations for the 65 documents that contain at least one verb form in -*si* (-*s̱i*),18CKD 7; 46; 63; 69; 83; 86b; 106; 107; 113; 128; 133; 140; 144; 157; 160; 161; 165; 201; 206; 211; 217; 223; 272; 283; 292; 302; 305; 309; 313; 317; 356; 357; 358; 361; 373; 376; 385; 387; 399; 414; 433; 448; 450; 526; 538; 546; 547; 552; 553; 578; 624; 625; 634; 635; 639; 663; 666; 696; 719; 721; 722; 775; 797; 819; 840. one can observe the following. 18 documents lack or do not preserve delivery instructions and are thus not helpful for this research.19CKD 69; 86b; 128; 201; 211; 283; 302; 313; 361; 376; 387; 414; 448; 578; 624; 625; 663; 775. Hence, we are left with a corpus of 47 documents, out of which 38 (∼81%)20CKD 7; 46; 63; 83; 107; 113; 133; 144; 161; 206; 217; 223; 272; 292; 305; 309; 317; 356; 357; 358; 373; 385; 399; 526; 538; 546; 547; 553; 634; 635; 639; 666; 696; 719; 721; 722; 797; 840. address only one person, as is expected for the 2sg ending -*si* (-*s̱i*). The remaining 9 documents (∼19%)21CKD 106; 140; 157; 160; 165; 433; 450; 552; 819. address more than one individual.

In documents addressing more than one person, it is often the case that a 2sg verb form is only intended for one person specifically.22The same has been observed for the recently discovered Bactrian documents, on which Sims-Williams (2007: 48) makes the following remark: “An alternation between 2 sg. and 2 pl. forms is very characteristic of the style of letters addressed to more than one person. In some such cases one might suppose that the writer has momentarily forgotten to whom he is writing, in others that he addresses a certain remark or command to one of his correspondents in particular”. I will briefly discuss two examples of this phenomenon. A first instance comes from CKD 140, a letter which is sent to the *ṣoṭhaṃgha* ‘accountant’ Lýipeya and three of his sisters. Being a *ṣoṭhaṃgha*, Lýipeya is concerned with the finances of the kingdom (cf. Burrow 1937: 127–128), while his sisters are not. As a result of this, when Kupṣiṃta, the author of CKD 140, makes a reference to the reckoning of taxes, it is most likely that he only addresses Lýipeya and not his sisters and, indeed, in those cases a 2sg verb form is used.23Interestingly, there is also a sentence in CKD 140 in which only one of the sisters is addressed (note the 2sg pronoun *tahi* ‘you.gen/dat.sg’): ***tahi Lýimsuas̱a*** *eda karyami cita kartavo* ‘**By you, Lýimsu,** attention is to be paid to this matter.’ (Burrow 1940: 25; emphasis mine). See example sentence (2), which occurs thrice (with minor variants) in CKD 140.

(2)

**Table:** 

***tuo***	*(puna)*	*ga(ṃ)nana*	***ja(ṃ)nas̱i***
you.dir.sg	again	reckoning.dir.sg	know.prs.2sg
‘**You** (again) **know** the reckoning of it.’			
(Burrow 1940: 25).			

Another telling example is found in CKD 552. This document is addressed to the same *ṣoṭhaṃgha* ‘accountant’ Lýipeya, this time together with his two scribes (*divira*) Sodaya and Lýimsu. In example sentence (3), again having to do with the administration of the Shanshan kingdom, the usage of a 2sg personal pronoun *tuo* ‘you’, combined with a nominal subject *bhaṭarag̱a* ‘master’ standing in apposition with the pronoun (*cf. supra* for this construction), makes it clear that again only Lýipeya is invoked here.

(3)

**Table:** 

***tuo***	***bhaṭarag̱a***	*mahi*	*jaṃna*	*nagaraṃmi*
you.dir.sg	master.dir.sg	me.gen/dat.1sg	people.dir.pl	city.loc.sg
***picavides̱i.***				
hand.over.pst.2sg				
‘**You the master have handed over** to me people (to be sent) to the city (?).’^24^				
(Burrow 1940: 109)				

24 The reading and interpretation of *nagaraṃmi* ‘in the city’ are not fully certain, but my argument is independent of the exact interpretation of this word.

For the ending -*tu* (-*du*), there is a total of 133 documents which contain at least one verb form with this ending,25CKD 1; 3; 4; 6; 7; 9; 11; 12; 15; 18; 20; 21; 24; 27; 29; 31 + 764; 32; 33; 36; 37; 39; 45; 47; 49; 53; 61; 62; 68; 71; 83; 86; 100; 106; 119; 124; 125; 126; 134; 135; 152; 157; 159; 162; 165; 177; 192; 206; 212; 217; 223; 235; 240; 244; 247; 248; 262; 265; 272; 278; 286; 292; 295; 297; 307; 308; 312; 317; 320; 329; 338; 341; 344; 357; 358; 362; 364; 367; 370; 373; 386; 393; 399; 408; 423; 438; 439; 450; 473; 475; 479; 480; 481; 482; 484; 492; 503; 509; 517; 519; 526; 528; 530; 538; 542; 545; 546; 548; 551; 554; 555; 562; 585; 606; 633; 634; 635; 636; 639; 714; 719; 720; 721; 722; 729; 734; 736; 738; 741; 761; 794; 796; 815; 819. out of which 6 are either not well enough preserved to be used for this investigation or simply do not indicate to whom they are addressed (CKD 125; 177; 240; 423; 741; 761). This leaves us with 127 documents. 75 of those (∼59%)26CKD 1; 4; 7; 9; 12; 15; 18; 20; 21; 24; 27; 29; 31 + 764; 32; 33; 36; 53; 61; 83; 86; 100; 135; 152; 206; 212; 217; 223; 235; 244; 248; 262; 272; 286; 292; 295; 297; 307; 312; 317; 329; 338; 341; 357; 358; 364; 367; 370; 373; 386; 393; 399; 408; 473; 479; 480; 484; 517; 526; 530; 538; 542; 545; 546; 548; 551; 555; 562; 585; 634; 635; 639; 719; 736; 738; 794. have only one individual as their addressee, while 52 of them (∼41%)27CKD 3; 6; 11; 37; 39; 45; 47; 49; 62; 68; 71; 106; 119; 124; 126; 134; 157; 159; 162; 165; 192; 247; 265; 278; 308; 320; 344; 362; 438; 439; 450; 475; 481; 482; 492; 503; 509; 519; 528; 554; 606; 633; 636; 714; 720; 721; 722; 729; 734; 796; 815; 819. have more than one person addressed. In other words, it is still the case that verb forms in -*tu* (-*du*) are found more often in letters and commands sent to only one person, but the difference with verbs ending in -*si* (-*s̱i*) is still notable, as for this ending 81% were addressed to only one person. The percentages we have for -*tu* (-*du*) are also not too far from the fifty-fifty ratio which we observed for the ending -*tha*, commonly believed to be plural.28In order not to give the impression of being biased, I have followed the suggestion of both reviewers to treat verb forms in -*tu* (-*du*) as a unified whole for these statistics. Yet, the reader should know that the statistics are partially influenced by stock phrases as *paribujiśatu* (*bujiśatu/paribhujiśatu/paribujiśadu*) ‘you will understand’ and *hotu* (*hodu*/*hutu*/*hoṃtu*/*bhavetu*) ‘it should be’ which were likely used without really paying attention to how many people are referred to in reality. In the case of *hotu* (*hodu*/*hutu*/*hoṃtu*/*bhavetu*), one should moreover reckon with the fact that this form is in most cases still synchronically analysable as a 3sg imperative (for which *cf. supra* and also Section 3.4). This is, for instance, the case in the expression *tahi zenig̱a ho(ṃ)tu* ‘he should be under your (sg) care’ (CKD 362; 370; 585; 721), where the third person subject can usually be inferred from a previous sentence or sentences in the document. When only counting documents that contain at least one other verb form -*tu* (-*du*) besides this type of stock phrases, there are 21/43 (∼49%) documents with verb forms in -*tu* (-*du*) addressed to only one person and 22/43 (∼51%) to more than one person. If these statistics are adopted, -*tu* (-*du*) would be even more in line with -*tha*. As a result, I take these numbers as another indication that the ending -*tu* (-*du*) is marked for 2pl, but that it can at the same time also be used as a polite form when referring to one person. As noted above, this same usage as a polite form seems to be there for the ending -*tha* as well.

While the figures discussed above and also summarized in Table 10 already suggest that the ending -*tu* (-*du*) is marked for plural, there are a few additional arguments to back this up. First of all, it is instructive to have a look at sentences which contain both a verb form in -*tu* (-*du*) and a form in -*si* (-*s̱i*). Such an example is, for instance, found in CKD 165, which is a personal letter sent by the *ogu*-official Kirtiśarma to both the *cozbo*-official Kranaya and the same *ṣoṭhaṃgha* Lýipeya which we encountered above. Example sentence (4) comes from this document and contains both the verb forms *vis̱ajiṣyatu* ‘you will send’ and *vyoṣiśas̱i* ‘you will reimburse’. If the ending -*tu* (-*du*) is indeed marked for plural, the assumption would be that *vis̱ajiṣyatu* would refer to both Kranaya and Lýipeya and 2sg *vyoṣiśas̱i* to only one of them. This suspicion can be easily confirmed, because *vyoṣiśas̱i* is combined not only with the 2sg pronoun *tuo*, but also by a nominal apposition, *ṣoṭhaṃgha Lýipeya*, which is akin to a vocative. As such, while *vis̱ajiṣyatu* can be taken to refer to more than one person, *vyoṣiśas̱i* only refers to one person in particular.29Incidentally, in another sentence from this same document, a certain comment is directed only to the *cozbo*-official Kranaya: ***tahi cozbo Kranayas̱a*** *lihami eda karyami* ***tuo*** *cita kartavya* ‘**To you *cozbo* Kranaya** I write. **You (** **sg** **)** must pay attention to this matter.’ (Burrow 1940: 32). Note the 2sg pronouns gen/dat.sg *tahi* and dir.sg *tuo*.


**Table 10:** People addressed in documents containing second person verb forms.

Verbal ending	Amount of well-preserved doc. mentioning the addressees	Amount of doc. with 1 individual addressed	%	Amount of doc. with more than 1 individual addressed	%
-*si* (-*s̱i*)	47	38	81	9	19
-*tu* (-*du*)	127	75	59	52	41
-*tha*	6	3	50	3	50

(4)

**Table:** 

*yati*	*tade*	*purima*	*pac̄ima*	***vis̱ajiṣyatu***	
if	this.abl.sg	earlier	later	send.fut.2pl	
*paṃthaṃmi*	*paras̱a*	*bhaviṣyati,*	*tuo*	***ṣoṭhaṃga***	
road.loc.sg	loss.dir.sg	be.fut.3sg	you.dir.sg	accountant.dir.sg	
***Lýipeya***	*tanu*	*goṭhade*	***vyoṣiśas̱i***		*nadhana*
Lýipeya.dir.sg	own.dir.sg	farm.abl.sg	reimburse.fut.2sg		parcel.gen.pl
*bhag̱ena*					
portion.ins.sg					
‘If **you (** **pl** **) send** it either earlier or later than then and it gets plundered on the way, **you (** **sg** **), *ṣoṭhaṃga* Lýipeya, will pay** it from your own farm, parcel for parcel.’					
(Burrow 1940: 32)							

A second point concerns the usage of second person verbs in the greeting formulae which open many letters in the Niya Prakrit corpus. One such formula translates into English as ‘I/we am/are happy that you are healthy’ and both *arog̱es̱i* and *arog̱etu* ‘you are healthy’ are found in this formula. What has not been observed before is that there is a distribution between these two forms. *arog̱es̱i* is found in CKD 302; 305; 385; 666; 721; 797; 840, all of which are addressed to only one person.30CKD 302 is only partially preserved, so for this one we cannot be sure.
*arog̱etu*, on the other hand, is attested in CKD 399; 714; 819. Two documents of this group (714; 819) are addressed to more than one person, while the remaining document (399) has a predilection for plural forms, as will be discussed in more detail *infra*. In addition, when *arog̱es̱i* and *arog̱etu* are combined with an overt personal pronoun in subject position, this pronoun is invariably *tuo* (*tuvo*/*tu*) ‘you.dir.sg’, while in CKD 714 *arog̱etu* is combined with *tus̱a* ‘you.dir.pl’ (for which see also below).31Speaking more generally, it is also the case that when *tusya* (*tus̱a*) ‘you.dir.pl’ is used in greeting formulae, this is always in a document that is sent to more than one person. See CKD 140; 475; 690. Taken together, these observations are further confirmation that the ending -*tu* (-*du*) should be seen as 2pl. Besides, one can now confidently restore the only partially preserved greeting formula of CKD 247 as ṣadosmi tusya arog(*etu) ‘I am pleased that you (pl) are healthy’, because of the pronoun *tusya* ‘you.dir.pl’ and the fact that this letter is addressed to two individuals (Cug̱apa and Priya[śaya]).

A third point worthy of note is that, with one exception (*vis̱arjitetu* ‘you have sent’ in CKD 399), all of the past tense forms ending in -*tu* (-*du*) are found in documents that are addressed to more than one person (CKD 126; 157; 162; 320; 519; 714).

As has been noted above, it seems likely that the ending -*tu* (-*du*) could have been used as a polite form as well. In some other cases, 2pl and 2sg may merely have been confused, for which parallels exist in Niya Prakrit.32In addition, compare again Sims-Williams’ (2007) remarks on Bactrian letters quoted in fn. 22. One sees such mistakes with the pronouns and verb endings of the first person too. A notable instance of such confusion is found in the introduction to CKD 475, here printed as example sentence (5). While no less than three people, named Yapg̱u, Cimg̱ayae and Parsug̱eya, are authoring this document, they still use 1sg verb forms and, strikingly, they combine the 1pl pronoun *veyaṃ* ‘we’ with a 1sg verb form *arog̱emi* ‘I am healthy’.33This particular example is paralleled in CKD 399 *veyaṃm* … *arog̱osmi* ‘we (!) … am healthy’.

(5)

**Table:** 

*tenaṃ*	*ca*	*suṭha*	ṣat(*o)smi		*yo*
this.ins.sg	and	very	be.pleased.prs.1sg		that.dir.sg
*tusya*	*arog̱a*	***śrudemi***	***veyaṃ***	*c(a)-iśa*	(*…*)
you.dir.pl	health.dir.sg	hear.pst.1sg	we.dir.pl	and-here	(…)
***arog̱emi***					
be.healthy.prs.1sg					
‘And therefore, **I am** very **pleased** that **I heard** you are healthy. Here too **we (!) am healthy** …’					
(tr. mine)					

Confusion between sg and pl forms can also be observed in nominal morphology. In the first part of CKD 399, for instance, one *cozbo*-official, named Ṣamas̱ena, is greeted by a string of honorific epithets, some of which are in the expected gen.sg (e.g. *priyadarśanasya* ‘having a dear look’), while others are gen.pl (e.g. *priyadevamaṃnuṣyana* ‘dear to gods and humans’). Addressing this *cozbo* Ṣamas̱ena with plural forms also continues in the rest of the document, where we find 2pl pronouns as *tumahu* and *tusmag̱a* and, interestingly, verbs ending in -*tu* (-*du*).

In view of the preceding arguments, it is now fair to conclude that the ending -*tu* (-*du*) marks 2pl in Niya Prakrit and not 2sg, as previously thought. As a result, Burrow’s paradigms of the present and the past tense, which were given above in Tables 4 and 5 respectively, should now be adapted. In Tables 11 and 12, the new paradigms are given with the differences indicated in bold face. Note that, in accordance with the absence of any attestation of the ending -*tha* for present tense, I have left it out of the paradigm for the present. I have further also included the possible translation of present forms ending in -*tu* (-*du*) as imperatives.

**Table 11:** Niya Prakrit present tense according to this study.

1sg	*denami* ‘I give’
2sg	*denasi* (*denas̱i*) ‘you (sg) give’
3sg	*denati* (*denadi*) ‘he/she gives’
1pl	*denama* ‘we give’
2pl	***denatu* (*denadu*) ‘you (** **pl** **) (should) give’**
3pl	*denaṃti* ‘they give’

**Table 12:** Niya Prakrit past tense according to this study.

1sg	*ditemi* ‘I gave’
2sg	*ditesi* (*dites̱i*) ‘you (sg) gave’
3sg	*dita* ‘he/she gave’
1pl	*ditama* ‘we gave’
2pl	*ditetha*; ***ditetu* (*ditedu*) ‘you (** **pl** **) gave’**
3pl	*ditaṃti* ‘they gave’

### 3.4 The etymology of the verbal ending -*tu* (-*du*)

As an offshoot of the synchronic description, this subsection presents a few stray remarks on the historical explanation of the ending -*tu* (-*du*). Various, often mutually exclusive, proposals have been made on the etymology of this verbal ending in Niya Prakrit. Thomas (1934: 49, fn. 5; 51, fn. 3; fn. 4; 57, fn. 1), for instance, presented no less than three different hypotheses on the origin of the ending -*tu* (-*du*). One suggestion of his is that this ending would be connected to the absolutive (“gerund”) in -*tu*, which has been observed in various Aśokan Prakrits (cf. Bloch 1950: 79). Yet Niya verbs ending in -*tu* (-*du*) do not have the meaning of an absolutive and absolutives in Niya Prakrit normally end in -*ti*.34Note that I interpret CKD 399 *vacitu* as a present form in -*tu* with the sense of an imperative, i.e. ‘you should read’ and not as an absolutive (see above). Another proposal by Thomas was to derive -*tu* (-*du*) from the OIA mediopassive imperative ending 3sg -*tām*, but this etymology is phonologically unlikely. It remains moreover unclear why Thomas (and, with him, Konow 1938: 155) considers these forms in -*tu* (-*du*) to be passive in origin, as *davyatu* ‘it should be given’ (CKD 399) is in fact the only example of a morphological passive with the ending -*tu* (-*du*).

The most straightforward of Thomas’ etymological suggestions is that -*tu* (-*du*) comes from the OIA active imperative ending 3sg -*tu*, derivatives of which are widely found in MIA languages (e.g. in Pāli; cf. Oberlies 2019 [2001]: 401–402). This etymology is not only phonologically attractive, but it also offers us an explanation as to why present tense forms in -*tu* (-*du*) can also express a command in Niya Prakrit. When taking the pragmatic context into account, one can moreover comprehend how such a third person ending could have been re-analysed as second person.35See in addition also the cross-linguistic remarks by Heine and Song (2010: 134–136, 2011: 601–602) on diachronic shifts from third to second person deixis in e.g. German.


In this regard, it is useful to take a brief look at two passages from the Pāli canon where such an imperative in -*tu* is used parallel to a second person imperative. A first example comes from the final verse of the *Punabbasusutta* contained in the Pāli *Saṃyuttanikāya* (SN I 210) – here printed as example sentence (6) – where a *yakṣa* mother is addressing her two children, using a second person imperative for her son Punabbasu and a third person imperative for her daughter Uttarā.

(6)

**Table:** 

***Punabbasu***	*sukhī*	***hohi.***
Punabbasu.voc.sg	happy.nom.sg	be.imp.2sg
*ajja-aham-hi*	*samuggatā.*		*diṭṭhāni*
today-I.nom.sg-indeed	emerge.pst.ptcp.nom.sg		see.pst.ptcp.nom.pl
*ariyasaccāni.*	***Uttarā***	*pi*	***suṇātu***
noble.truth.nom.pl	Uttarā.nom.sg	also	listen.imp.3sg
*me-ti.*
me.gen.sg.^36^-thus
‘**Punabbasu, be** happy! Today I have emerged at last. **Hear** me too, **O Uttarā**: The noble truths are seen!’			
(Bodhi 2000: 311)			

36 Even though formally *me* can be both acc.sg and gen.sg, I take it here to be gen.sg, because the verb *suṇāti* ‘to hear’ takes the genitive case for the person to whom one listens. See Peterson (1998: 100; 103–104).

Even though the *yakṣa* mother uses the third person imperative *suṇātu*, second person deixis is implied, because we are dealing here with a speech act whereby person A, in this case the mother, is directly addressing person B, in this case the daughter. This point is nicely captured by Bhikkhu Bodhi’s translation, as he renders both the 2sg *hohi* and the 3sg *suṇātu* as a second person imperative in English.37In the first verse of this *Punabbasusutta*, the mother addresses both the daughter and the son with the second person imperative *hohi*: *Tuṇhī uttarike* ***hohi*** *tuṇhī* ***hohi*** *punabbasu, yāvāhaṃ buddhaseṭṭhassa dhammaṃ sossāmi satthano* ‘**Be** quiet, Uttarikā, **be** quiet, Punabbasu! I wish to listen to the Dhamma of the Teacher, the Supreme Buddha.’ (Bodhi 2000: 310). There is no metrical reason why 3sg *suṇātu* ‘one should hear’ was adopted in example sentence (6), as 2sg *suṇāhi* ‘listen’ would be metrically equivalent. Letters and royal commands represent this same type of speech act whereby person A is addressing person B (C, D etc.), which in turn makes it understandable that a third person verb form could have become second person in Niya Prakrit.38Given that the endings of the nominative and the vocative case are often the same in MIA, the subject of such a third person imperative can also have been re-analysed in some cases as a vocative.


It should still be admitted that *suṇātu* is paired together with a second singular imperative *hohi* in example sentence (6), whereas I assume this ending -*tu* to have become a second plural ending in Niya Prakrit. That this is indeed possible can be seen from another passage of the Pāli canon, this time from the *Mahāvagga* (II, 3.3 = Vin. I 102–103). The excerpt from the *Uposathakkhandhaka* of the *Mahāvagga* in (7) is concerned with the recitation at the 15th day of the month (the *Uposatha*-day) of the *Pātimokkha*-rules Buddhist monks have to comply with.

(7)

**Table:** 

*vyattena*	*bhikkhunā*	*paṭibalena*	*saṃgho*	
experienced.ins.sg	monk.ins.sg	competent.ins.sg	community.nom.sg	
*ñāpetabbo.*	***‘suṇātu***	*me*	***bhante***
inform.ger.nom.sg	listen.imp.3sg	me.gen.sg.	venerable.voc.
*saṃgho.*	(*…*)	*kiṃ*	*saṃghassa*
community.nom.sg	(…)	what	community.gen.sg
*pubba-kiccaṃ?*	*pārisuddhiṃ*	***āyasmanto***	***ārocetha.***	
principal.duty.nom.sg	purity.acc.sg	venerable.voc.pl	announce.imp.2pl	
*pātimokkhaṃ*	*uddisissāmi.*
Pātimokkha.acc.sg	recite.fut.1sg
‘The Order should be informed by an experienced, competent monk, saying: ‘**Honoured sirs, let** the Order **listen** to me. (…) What is the Order’s first duty? **Let the venerable ones announce** entire purity. I will recite the *Pātimokkha* …’’				
(Horner 1951: 132)				

The introductory sentence makes it clear that a senior, experienced monk should address the community of Buddhist monks.39For a recent discussion on the historical background of the passage cited, see Wynne (2020: 192–194). As the Buddhist *sangha* [monastic order] consists by necessity of several monks, this means that 3sg *suṇātu* in *suṇātu me bhante saṃgho* ‘the *sangha*, venerable ones, should listen to me’ is pragmatically comparable to a 2pl imperative of the type ‘listen to me, monks’. Therefore, this usage of *suṇātu* by the senior monk can be compared to the Buddha’s use of a 2pl imperative *suṇātha* ‘listen’ in a stock phrase of the Pāli canon used to address the monks, here example (8).

(8)

**Table:** 

*tena*	*hi*	***bhikkhave***		***suṇātha***	*sādhukaṃ*
this.ins.sg	indeed	monk.voc.pl		listen.imp.2pl	well
***manasikarotha***		*bhāsissāmī-ti.*			
pay.attention.imp.2pl		speak.fut.1sg-thus			
‘Therefore, **monks, listen** indeed. **Pay** (close) **attention**. I will speak.’					
(tr. mine)					

Further confirmation for the view that *suṇātu* more or less equals *suṇātha* can be drawn from what follows in the rest of the *Mahāvagga*-portion. Without there being a change in the type of speech act, the senior monk suddenly switches to the 2pl imperative *ārocetha* ‘announce’ in his address to the community of monks.

Using these Pāli examples as a *comparandum*, we can thus hypothesize that this type of third person imperatives came first to be re-analysed in the prehistory of Niya Prakrit as second person (plural) imperatives because of the pragmatic context is which they were used. The assumption would then be that, once the originally imperative ending -*tu* (-*du*) was re-interpreted as a second person (plural) ending, its usage got extended, so as to be also useable in declarative statements.40As the second person plural imperative and indicative ending are often the same in MIA languages (e.g. -*tha* is used for both in Pāli; see Oberlies 2019 [2001]: 402), one could conjecture that, once -*tu* (-*du*) had firmly established itself as a 2pl imperative ending, it was analogically introduced in the paradigm of the indicative as well.


On a more general level, there is a further reason why the ending in -*tu* could have been re-interpreted as a plural ending specifically. In the Pāli examples cited above, the imperative is constructed with a nominal subject, e.g. *Uttarā* … *suṇātu*, literally ‘Uttarā should listen’. Yet, this type of imperatives can also be used without an overt subject, e.g. *suṇātu* ‘one should listen’. In such cases, the agent is not always specified, because of which *suṇātu* can at times even mean ‘anyone should listen’. Typologists sometimes refer to this type of construction as a “non-referential indefinite” (e.g. Ramat and Sansò 2007) and what is relevant for us is that such a construction may come to be marked for plural number. This is so because different options are left open as the exact referent is not specified. In this respect, it is worth pointing to the so-called MAN-impersonals that are found in various European languages (see e.g. Ramat and Sansò 2007; Siewierska 2011). These MAN-impersonals are a type of impersonal construction whereby the indefinite pronoun in subject position is etymologically derived from a lexeme meaning ‘man’. An example is the French impersonal construction *on* + 3sg verb, e.g. *on verra*, which literally means ‘one shall see’. Incidentally, this construction with *on* is further grammaticalized in French, especially so in informal registers, to that of a 1pl, thereby gradually replacing the older construction with the 1pl personal pronoun *nous* ‘we’ + 1pl verb (cf. e.g. Ramat and Sansò 2007: 104–106; Heine and Song 2011: 616). In Danish, these MAN-impersonals may also refer to the second and third person (cf. Siewierska 2011: 65), in which regard they are closer than the French to what I assume for the ending -*tu* (-*du*) in Niya Prakrit.

## 4 Concluding remarks

The main results of the present paper can be summarized as follows. First, *tusya*/*tus̱a* ‘you’ is not another gen.sg of the second person pronoun, but the hitherto missing dir.pl. Etymologically, *tusya*/*tus̱a* likely derives from OIA *yūyam* ‘you.nom.pl’, whereby *yū*- has been analogically replaced by *tus*- from the oblique forms. Second, various observations suggest that the ending -*tu* (-*du*) is not a mere variant of the 2sg ending -*si* (-*s̱i*), but that -*tu* (-*du*) is in fact replacing the old 2pl verbal ending -*tha*. The latter ending was clearly in decline, as it is only found in past tense forms. In other words, -*tu* (-*du*) is not a 2sg ending as usually assumed, although it can also be used as a polite form. Given that the ending -*tu* (-*du*) can in addition still express commands in the present tense, it is likely that -*tu* (-*du*) etymologically derives from the OIA 3sg imperative in -*tu*.

Even when one may disagree with some parts of the analysis proposed here, it should at least be clear that the grammar of Niya Prakrit is by far not completely understood and is in need of careful examination. Possible topics for further research include, for instance, factors determining the choice between different imperatival moods and tenses (e.g. *tu*-forms vs. gerundives) or the syntax of relative clauses. In the end, one could then “draw up a new balance of all the invaluable things Niya teaches us”, as Caillat (1990: 10) already suggested thirty years ago.
